# Short Stature Diagnosis and Referral

**DOI:** 10.3389/fendo.2017.00374

**Published:** 2018-01-11

**Authors:** Mohamad Maghnie, José I. Labarta, Ekaterina Koledova, Tilman R. Rohrer

**Affiliations:** ^1^Department of Pediatrics, IRCCS Children’s Hospital Giannina Gaslini, University of Genoa, Genoa, Italy; ^2^Endocrinology Unit, Children’s Hospital Miguel Servet, University of Zaragoza, Zaragoza, Spain; ^3^Global Medical Affairs, Merck KGaA, Darmstadt, Germany; ^4^Department of Pediatrics, Saarland University Medical Center, Homburg, Germany

**Keywords:** growth hormone, short stature, diagnosis, referral, patient management

## Abstract

The “360° GH in Europe” meeting, which examined various aspects of GH diseases, was held in Lisbon, Portugal, in June 2016. The Merck KGaA (Germany) funded meeting comprised three sessions entitled “*Short Stature Diagnosis and Referral*,” “*Optimizing Patient Management*,” and “*Managing Transition*.” Each session had three speaker presentations, followed by a discussion period, and is reported as a manuscript, authored by the speakers. The first session examined current processes of diagnosis and referral by endocrine specialists for pediatric patients with short stature. Requirements for referral vary widely, by country and by patient characteristics such as age. A balance must be made to ensure eligible patients get referred while healthcare systems are not over-burdened by excessive referrals. Late referral and diagnosis of non-GH deficiency conditions can result in increased morbidity and mortality. The consequent delays in making a diagnosis may compromise the effectiveness of GH treatment. Algorithms for growth monitoring and evaluation of skeletal disproportions can improve identification of non-GH deficiency conditions. Performance and validation of guidelines for diagnosis of GH deficiency have not been sufficiently tested. Provocative tests for investigation of GH deficiency remain equivocal, with insufficient information on variations due to patient characteristics, and cutoff values for definition differ not only by country but also by the assay used. When referring and diagnosing causes of short stature in pediatric patients, clinicians need to rely on many factors, but the most essential is clinical experience.

## Introduction

A pediatric patient with short stature and no clinically evident reason generally requires referral to an endocrine specialist to identify the cause. The subsequent diagnosis may include one of several conditions in which GH therapy has been approved for use. Guidelines for referral vary widely, but should be sufficient to enable patients to receive appropriate treatment without over-burdening the healthcare systems. For optimal efficacy of GH treatment, the appropriate diagnosis should be made and treatment initiated as early as possible in the life of the patient (1). Published guidelines for diagnosis of conditions that require GH therapy are not universal or free from inaccuracies, varying with both the immunoassays used (2, 3) and clinical factors such as the age and body mass index (BMI) of the patient (4, 5). Cutoff values of GH provocation tests used to determine whether a patient receives GH treatment also vary according to country-specific regulations and socioeconomic conditions. While the majority of short stature cases treatable with GH involve idiopathic GH deficiency, the cause must be identified as specifically as possible and there are a number of non-GH deficiency short stature conditions that have approval in various countries and for individual GH formulations (6, 7). The following report summarizes the first of the three sessions from a meeting held in Lisbon, Portugal, funded by Merck KGaA (Germany), which aimed to examine aspects of diagnosis and referral for GH treatment. The reports from the sessions on optimizing patient management and on transition from pediatric to adult care are published in accompanying articles (8, 9).

## Clinical Indications for Referral for Short Stature

Short stature can be due to various etiologies and the cause may be a primary or secondary growth disorder, or idiopathic (10). Primary growth disorders are intrinsic to the growth plate and include clinically defined syndromes, factors that result in being born small for gestational age (SGA), and skeletal dysplasias. Secondary growth disorders are believed to change the milieu of the growth plate and include GH deficiency, disorders of the GH–insulin-like growth factor (IGF)-I axis including IGF-I deficiency or resistance, endocrine and metabolic disorders, organ system disorders, malnutrition, psychosocial disorders, and iatrogenic conditions. Patients with idiopathic short stature (ISS) have no discernible cause, the condition is very heterogeneous and may be either familial or non-familial. In all cases, an early diagnosis is important and, therefore, height screening programs must be sufficiently sensitive and specific to ensure timely detection and treatment (11). The prevalence of pathological cases among children referred for short stature has been reported to range from 1.3 to 19.8% (11), depending on the criteria for referral. There are no indicators to suggest that pathological causes of growth failure have different prevalence in different countries, except for growth failure caused by malnutrition, which is obviously dependent on socioeconomic circumstances.

After exclusion of clinical conditions that may result in short stature, a diagnosis of the cause of growth failure and potential need for GH treatment should be made by an endocrine specialist. Such patients require a referral, which may come from a variety of sources within the healthcare system. Guidelines for referral vary in different countries (12), but strict application of criteria may lead to unnecessary referrals that over-burden the healthcare system (13). A comparison of Dutch, Finnish, and UK guidelines, based on height standard deviation score (SDS), height SDS in relation to target height SDS, and height SDS deflection, found sensitivities of 74, 78, and 57% for the respective guidelines when using all three criteria (Table 1) (14). However, the study indicated that up to 25% of children with pathological growth failure may not be identified and other factors need to be considered, such as clinical examination and phenotype. There is a strong gender bias in referrals for short stature, with girls much less likely to be referred than boys (15, 16) and an ethnic bias may also be present (16). In all cases, up-to-date growth standards should be used, and wherever possible they should be population specific. A study of Finnish girls with Turner syndrome found a greatly increased sensitivity to detect the condition when using population-specific Finnish standards compared with use of WHO standards (17). Similarly, a study of USA children with cystic fibrosis at age 2 years found growth failure in 9% when using WHO standards compared with 26% when using USA CDC prevention standards (18).

**Table 1:** Comparison of country-specific guidelines for referral of short children for diagnostic work-up.

Auxological criteria	Dutch guideline			Finnish guideline			British guideline		
	Cutoff	Sensitivity (%)	Specificity (%)	Cutoff	Sensitivity (%)	Specificity (%)	Cutoff	Sensitivity (%)	Specificity (%)
Height SDS	<−2.5	48	99.1	<−2.2	74	98.4	<−2.7	30	99.4
Height SDS–target height SDS	<−1.6	70	99.1	<−2.2	61	94.4	<−2.0	39	99.0
Height deflection	>1.0 SD	4	99.9	>2.2 SD	9	88.0	>1.3 SD	4	97.1
Any of the above criteria		74	98.5		78	83.7		57	95.8

*Adapted from data reported by Stalman et al. (14)*.

The algorithms required for referral for diagnostic work-up in cases with short stature differ according to the age of the patient (19, 20). For patients less than 3 years old, phases of catch-up growth occur and repeated measurements of height SDS are necessary. At that age, height measurements have high variability and less accuracy, and the decision rules based on distance from target height and/or height deflection have low-predictive value; therefore, referral should be based on severe short stature (height SDS <−3) or repeated measurements (height SDS <−2.5 repeated within 1 year). For patients older than 3 years, in addition to height SDS, multiple factors should be taken into consideration, such as a history of being born SGA, body disproportion, dysmorphic features, emotional deprivation or subnormal growth velocity, and distance from target height. However, such algorithms are only diagnostic tools and clinical judgment must be used in all cases.

Efficacy of GH treatment is better when started at a young age, and diagnosis should, therefore, be made as early as possible. Diagnostic delay was retrospectively examined in a cohort of 21 patients with pituitary stalk interruption syndrome and GH deficiency (21). The median age at diagnosis was 3.6 years, but examination of auxological criteria, as defined by the GH Research Society (22), indicated a median delay in diagnosis of 2.3 years. The most effective criterion was height SDS more than 1.5 below target height SDS, with 90% of the patients meeting this criterion at median age of 1 year (21).

Similarly, a delay in diagnosis of children with celiac disease and associated growth failure was observed (23); the study found that growth monitoring could have identified children with abnormal growth 2–3 years prior to the diagnosis of celiac disease. However, no single factor provided sufficient sensitivity and a combination of auxological factors was required. A delay in diagnosis of children with Turner syndrome has also been shown, and a combination of factors was also found to enable identification at an early age (24, 25). The age of diagnosis of Turner syndrome is typically older than 10 years, with 22% of cases diagnosed at age >12 years; using factors of height SDS <−2, height SDS minus target height SDS <−2, and growth rate could have identified approximately 80% of patients with short stature at age <5 years (25).

There is currently no consensus on which screening tests should be performed when evaluating children with short stature. The pathology detection rate in asymptomatic short children remains low. In a retrospective review of 1,373 referrals for short stature to a pediatric endocrine clinic, there were 235 children with height less than the third percentile who were normal on physical examination and had a normal growth velocity (>5 cm/year) with no abnormal medical history (26). Only three of these children had a newly diagnosed pathology, and almost 99% had no evident pathology on laboratory or radiological screening and were considered to be normal variant short stature, implying that there is a very low incidence of pathology in healthy short children growing at a normal rate. Of the three with pathology relating to short stature, one had IGF-I resistance and two had celiac disease, and celiac disease may be the underlying cause of short stature in 2–8% of cases, particularly when other causes are excluded.

Factors such as normal IGF-I and growth velocity >25th percentile reduce the likelihood of GH deficiency as the cause of short stature (27). Highly sensitive decision rules have been suggested to identify which patients require GH testing (28). Therefore, evaluations of such patients should be directed, based on medical history and careful clinical examination. Algorithms can be drawn up to enable identification of the causes of short stature based on body proportions, prenatal growth, and IGF-I levels (11, 29, 30). Such algorithms can be used to determine whether genetic testing is required to detect causes of short stature other than GH deficiency (29). Algorithms can also be integrated into electronic records for automated growth monitoring and are more efficient than standard monitoring, so they have become the preferred option in current clinical practice.

## Diagnosis and Referral for Non-GH Deficiency Disorders

Height screening programs in primary healthcare are not universal, even in developed countries, which frequently results in late diagnosis of growth disorders. In patients with Turner syndrome, late diagnosis at a median age of 15 years was found to be associated with increased mortality and incidence of comorbidities such as congenital heart defects and ovarian dysfunction (24). Conversely, children born SGA are more often diagnosed early, resulting in a longer duration of GH treatment and consequent increased height gain.

In order to improve referral, collaboration between primary and secondary healthcare providers should be promoted for monitoring of growth of children. In the Netherlands, guidelines for referral are based on screening parameters of height SDS, height SDS relative to target height SDS, and height SDS deflection, but result in approximately 38% of all children requiring referral (13). However, these evidence-based guidelines, based on an algorithm for diagnosis, are considered to provide the best sensitivity and specificity (11, 12). The algorithm includes a parameter of disproportion or dysmorphic features, which can indicate conditions such as short-stature homeobox (*SHOX*) gene insufficiency and Noonan syndrome.

Turner syndrome occurs in about 1 in 2,000 live-born girls and diagnosis is based on short stature, hypogonadism, and minor anomalies (31). Where an X chromosome is completely or partly missing, the condition may be associated with other anomalies due to *SHOX* haploinsufficiency (30). Diagnosis can occur prenatally, in newborns with lymphedema, in small children with short stature, at school age when associated with delayed puberty or, in rare cases, precocious puberty (32), or in adolescents with primary amenorrhea.

Skeletal disproportions can be detected by plotting the extremities-to-trunk ratio against height, with a deviation >−1 SD indicating SHOX protein deficiency. Noonan syndrome is an autosomal dominant inherited condition with prevalence of approximately 1 in 2,000 live births (33). The condition is associated with mutations in the *PTPN11* gene and other genes in the Ras/MAP kinase signaling pathway. Patients are characterized by facial dysmorphology, heart defects, and short stature, although GH is generally normal but GH sensitivity may be reduced.

Automated screening using electronic health records helps to improve referrals for such patients with short stature. In Finland, primary care nurses are trained in anthropometry techniques and population-specific growth references are available (34, 35). Screening rules are integrated into each child’s electronic records, with automatic transfer of abnormal values to a pediatric endocrinologist. Testing of this automated growth monitoring over a 1-year period resulted in an increase in prevalence of detection of growth disorders in the Finnish population from 5.9 to 13.4% of referred children (35). In Germany, between the years 2000 and 2005 the CrescNet program developed at Leipzig University enabled a reduction in age of identification of GH deficiency and earlier initiation of GH treatment (36). This systematic screening program now includes a large number of medical practices and pediatric endocrinology centers, and mean age at GH start has continued to decrease across the country.

The WHO growth standards are still used in approximately 125 countries, even though children in Finland were shown to be 0.2–0.8 SD taller. Thus, use of the WHO standards would miss about half of the patients with Turner syndrome (17). For screening of patients born SGA, who require monitoring for continued short stature, software systems using population-specific neonatal records are available (36–38). While laboratory evaluations for multiple factors such as renal function, liver function, thyroid hormone levels, karyotyping, and skeletal dysmorphism are available, laboratory testing without specific diagnostic questions is not cost-efficient (26). Ultimately, indiscriminate testing cannot replace clinical skills in evaluation of patients with short stature.

## Gaps in the Diagnosis of Short Stature Due to GH Deficiency

A patient who is identified as having short stature is referred to an endocrine specialist for investigation of the cause. The diagnosis is made from clinical and phenotype assessments with growth monitoring and laboratory evaluations. Among conditions with approval for GH therapy, the most frequently identified cause is GH deficiency and evaluation of GH secretory status is required. In cases with suspected hypopituitarism, hypothalamic–pituitary imaging is advisable to exclude tumors and identify ectopic and hypoplastic pituitary glands; targeted genetic analyses may also be carried out. For patients thought likely to respond to GH in terms of growth, treatment requires appropriate resources and facilities to be in place, which will differ by country and also by the socioeconomic environment for the particular patient situation. However, there appears to be a gender bias in GH treatment of children with short stature in many countries; males are much more likely to receive GH treatment, irrespective of the underlying diagnosis of the cause of short stature (15, 39).

Guidelines for initiation of endocrine investigation and for ensuring that children and adolescents with GH deficiency are identified appropriately were published by the GH Research Society in 2000 (22) and, more recently, by the Pediatric Endocrine Society (40). However, the performance of the guidelines and the level of validation have never been tested, and the guidelines start with the presumption that other causes of short stature, such as hypothyroidism and chronic systemic diseases, have been excluded. A recent systematic survey (19) of more than 1,500 publications with reported algorithms for growth monitoring found no consensus on conditions that should be targeted. The study identified six conditions that had been targeted (Turner syndrome, celiac disease, cystic fibrosis, GH deficiency, renal tubular acidosis, and SGA), but standardization of detection tools was very low, none of the studies had internal validation, and external validation was often incomplete. The information implied that the evidence supporting growth monitoring is suboptimal in most countries, which results in delayed diagnosis of severe disorders and inappropriate referrals of children with normal variants of growth. Therefore, while early detection of underlying causes of growth disorders is warranted, there remains a need for identifying priority target conditions and appropriate detection tools.

A survey among pediatric endocrinologists around the world, and specifically members of the European Society of Pediatric Endocrinology, showed that growth monitoring was very variable and laboratory investigations for diagnostic work-up were frequently not applied in routine clinical practice (12). Laboratory determinations to identify cases of GH deficiency include GH stimulation tests, but these need to be carried out appropriately. In a study designed to characterize possible causes of false-positive diagnoses of GH deficiency, the effects of calorie intake were examined (41). Insulin or arginine stimulation tests were carried out before and after 3 days on a hypocaloric diet. The results showed that GH response was significantly increased after the hypocaloric diet. The strong influence of diet clearly contributes to poor reproducibility of stimulation tests and a high incidence of false positives.

In very young children, the GHRH-arginine test may not conclusively differentiate GH deficiency from normal (42) and the insulin tolerance test may result in hypoglycemia due to high sensitivity to insulin (43). Accuracy of the glucagon test was examined in a study of children younger than 6 years who had GH deficiency confirmed by peak GH less than 10 µg/l in insulin and arginine tests (44). The mean GH response to glucagon was significantly greater than both the insulin and arginine tests, indicating confusing diagnostic information depending on which of the current stimulation tests is used. The peak GH response to glucagon was correlated inversely with age of the patient, although not with BMI. However, other studies have shown that BMI significantly affects response to GH stimulation tests, with reduced peak GH seen in obese children (5). Therefore, normative data for each stimulation test according to age, sex, and BMI are required.

To define GH deficiency, a cutoff for peak stimulated GH concentration below 10 µg/l was often used, as suggested by the GH Research Society (22). However, cutoff values of 3 or 5 µg/l are also used and the cutoff remains somewhat arbitrary (40, 45). In addition, the cutoff values for commercially available immunoassay-based GH stimulation tests have been shown to vary greatly, with little reference data available (2). A survey of 19 health centers in Germany found little agreement between results (46) and an audit of current practices in Europe suggested that cutoffs were based on tradition rather than assay characteristics (47). In another study, cutoffs of 4.3–7.8 µg/l were found for different assays, against a value of 7.1 µg/l for an automated system validated against isotope-dilution mass spectrometry (3). Thus, clinicians should ensure that they are familiar with the assay being used in their local laboratory and should be aware of the impact of factors such as age, nutritional status, BMI, sex, and pubertal stage.

As well as biochemical tests, magnetic resonance imaging (MRI) of the central nervous system is often used to classify patients with GH deficiency. In a large observational study of patients receiving GH treatment, there were 1,844 with a diagnosis of either ISS or born SGA (48). Pituitary abnormalities were found on MRI in 151 of these patients, with pituitary hypoplasia observed most frequently. Ectopic posterior pituitary was found in 20 of the patients, which were equally distributed between the diagnoses of ISS and SGA. This result suggested that there was misclassification of patients based on the GH response to stimulation testing. In a recent anecdotal case, a boy aged 8 years and 8 months was diagnosed with GH deficiency at age 3 years, based on short stature and severe hypoglycemia. An earlier MRI showed no bright spot of the posterior pituitary, but no further action was taken because no evidence of diabetes insipidus was found. Examination of a second MRI showed that the patient had anterior pituitary hypoplasia, pituitary stalk agenesis, and ectopic posterior pituitary at the level of median eminence, which was incorrectly measured as a 3-mm-thick pituitary stalk (personal communication). Thus, MRI pitfalls in the diagnosis of structural pituitary abnormalities in inexperienced hands suggest that MRI is a factor that should be examined very carefully when making a diagnosis in a patient with short stature.

## Discussion Session

Identifying the need to treat patients with GH remains challenging in a number of conditions, for instance IGF-I resistance. In such cases, the clinician must first rule out GH deficiency and then assess the status of the IGF receptor, if considered necessary. Algorithms for identifying growth disorders generally assume normal weight, but an abnormal BMI can be used to assess nutritional disorders or conditions such as cystic fibrosis. In order to improve diagnosis of short stature in patients with Turner syndrome, healthcare practitioners need to be given better criteria for testing, and factors such as delayed puberty need to be taken more seriously. Clinicians also need guidelines to identify the need for referral for children with suspected Prader–Willi syndrome. An international consensus has been reported (49) and it was noted that in Germany there are published national criteria and a methylation test is always performed in suspected cases.

Small for gestational age is a heterogeneous condition and the term “children born SGA” is essentially a description and not strictly a diagnosis. The diagnosis of GH deficiency and pituitary dysfunction may be missed in a number of patients (48) and there is a high frequency of pituitary defects, leading to the question of whether MRI should be used routinely in SGA. In general, it would be recommended to look at the patient’s phenotype and characteristics in cases of suspected GH deficiency and at IGF-I status before performing an MRI. However, GH is often initiated without sufficient testing, where patients may have conditions such as a mid-line defect, and there are protocols to identify individuals who should have MRI assessment. Not all children born SGA are followed up, and only children born less than 1,500 g are routinely followed.

There remain defects in testing for GH deficiency and WHO charts are not good for diagnosis in some conditions associated with short stature. It was noted that currently in Australia diagnosis of short stature is based on auxology, with limited laboratory testing (50), although there are no reports at present to indicate whether this is cost effective. However, auxology criteria are applied accurately and carefully in order to avoid over-treating of patients. Additionally, automated electronic records are available for use in Australia (51), as well as in Finland (35) and a number of other countries (19). National population reference charts are needed, although the WHO weight charts are good for identifying obesity in the first 2 years of life. WHO charts are used in Australia for the first 2 years after birth, but CDC charts are used subsequently; however, reference values may vary for specific ethnic groups.

Evaluation of GH deficiency in the first 1–2 years of life is difficult, since there is a lack of reference data. Some data do exist using random GH tests in newborns (52); however, patients in Germany may receive MRI testing if indicated. Genetic testing may also be carried out if short stature is detected early in life. Since the publication of the GH Research Society guidelines in 2000 (22), task forces have been trying to identify the best parameters for early diagnosis of GH deficiency. While guidelines have been revised to some extent (40), evaluation during very early childhood has not been adequately addressed and there remains a need for an update on consensus guidelines based on recent knowledge. In Germany, neonatal length and weight data are available (37); target height data are also determined and used with electronic data to provide automatic referral for short stature. Improved education is needed for all clinicians in order to provide better referral and diagnosis. The current need is to address the lack of links between primary and secondary medical facilities, and the different specialties involved.

In summary, children with short stature require referral to specialist centers for diagnosis of the cause of growth failure. Guidelines for referral vary by country and patient characteristics, but must be appropriate in order that healthcare systems are not over-whelmed. The diagnosis and, where necessary, treatment with GH should be initiated as early as possible. Algorithms have been proposed for identification of patients that may benefits from GH therapy for short stature, whether due to GH deficiency or non-GH deficiency conditions. However, performance and validation of guidelines for diagnosis have not been sufficiently tested; biochemical tests for GH deficiency remain equivocal, with inadequate information on effects of patient characteristics and definitive cutoff values. Clinicians need to rely on many factors for referral and diagnosis of causes of short stature in children, in order to provide the correct treatment with GH. Therefore, evaluations should be directed, based on medical history and careful clinical examination.

## Author Contributions

All authors critically revised the current work for important intellectual content and gave final approval of the version of the publication to be published. All authors have agreed to be accountable for all aspects of the work in ensuring that questions related to the accuracy or integrity of any part of it are appropriately investigated and resolved.

## Conflict of Interest Statement

MM reports grants and personal fees and lecture fees from University of Genova outside the submitted work. JL reports grants and personal fees from Merck outside the submitted work. EK is an employee of Merck KGaA. TR reports grants and personal fees from University of Saarland lecture fees outside the submitted work.
